# The Incidence of Retrograde Peri-Implantitis in a Single University Dental Hospital Training Center: A Retrospective Analysis

**DOI:** 10.3390/medicina59030560

**Published:** 2023-03-13

**Authors:** Lamees R. Alssum, Maha M. Alghofaily, Asrar S. Aleyiydi, Sadeem A. Alomar, Fahd M. Alsalleeh

**Affiliations:** 1 Department of Periodontics and Community Dentistry, College of Dentistry, King Saud University, P.O. Box 60169, Riyadh 11545, Saudi Arabia; 2 Department of Restorative Dental Sciences, College of Dentistry, King Saud University, P.O. Box 60169, Riyadh 11545, Saudi Arabia; 3 College of Dentistry, King Saud University, P.O. Box 60169, Riyadh 11545, Saudi Arabia

**Keywords:** peri-implantitis, complications, dental implants, endodontically treated teeth

## Abstract

*Background and objective*: Retrograde peri-implantitis (RPI) is a periapical radiolucent lesion developed around the implant apex. This study aimed to investigate the Incidence of RPI in a single university dental hospital training center. *Materials and Methods*: All records of patients who received single Implants between 2016–2020 were screened. For cases that met inclusion criteria, clinical and radiographic data were analyzed. *Results*: A total of 215 were included and categorized as follows, Category A: implants were placed next to endodontically treated teeth (*n* = 58, 27%); category B, implants placed at the sites with previous endodontic involvement within 6 months of tooth extraction (*n* = 25, 11.6%); Category AB: implants placed at sites that fulfill the criteria of groups A and B (*n* = 18, 8.4%); and Category C: Implants that were placed next to vital teeth and at a site with no previous endodontic treatment or a site that was allowed to heal for more than six (*n* = 114, 53%). Categories A, B and AB served as the endodontically involved (EI) group, while category C served as non- endodontically involved (NEI) group. Only two sites (0.9%) were confirmed as RPI, both from group A (3.4%). Comparing all groups studied showed no statistically significant difference in RPI incidence. *Conclusions*: The incidence of RPI is low; however, endodontically treated teeth with periapical lesions (PALs) next to an implant site could contribute to RPI.

## 1. Introduction

Dental implants are an excellent treatment option to replace single or multiple missing teeth, supporting fixed and removable prostheses [1,2]. It helps restore function and esthetic, providing ridge preservation and improving patient confidence and well-being. According to the European Association of osteointegration (EAO), the implant success rates 10 years after placement for single crowns and fixed dental prostheses are 94.9% and 92.8%, respectively [3].

Although dental implant treatment is highly successful, many factors can cause implant complications and subsequent failures [4]. It includes bone overheating during implant placement, surgical trauma, cortical bone perforation, and secondary site infection to aseptic technique. These factors could lead to implant osseointegration failure and infection of the peri-implant tissues, commonly seen in peri-implant mucositis or peri-implantitis [5,6]. Peri-implant mucositis is defined as an inflammatory lesion of the mucosa surrounding an endosseous implant without loss of supporting peri-implant bone. While peri-implantitis is a pathological condition occurring in tissues around dental implants, characterized by inflammation in the peri-implant mucosa and progressive loss of supporting bone [7]. Recently, several case reports and case series presented a new pathological disease entity, defining an infectious process that occurs around the apical part of the implant; Retrograde peri-implantitis (RPI) [8,9,10,11,12].

Retrograde peri-implantitis was first described in 1992 by McAllister et al. [13]. They presented two implant cases that showed radiographic changes around the implant apex with clinical signs of infection. Another case report came out soon after to document a similar infectious process around an implant. The case was described as localized osteomyelitis, and the implant was lost within the first few weeks [14]. Several terminologies were used to describe this infectious disease process, including periapical implant pathology, apical peri-implantitis, and RPI [15,16,17].

RPI is a clinically symptomatic periapical radiolucent lesion that develops shortly after implant insertion, while the coronal portion of the implant achieves a typical bone-to-implant interface [18]. Different hypotheses have been discussed as possible etiologies for RPI. It can be associated with biological change or mechanical mishaps before, during or after implant placement [14,19]. Most commonly, it is associated with infection from the same site after tooth extraction or adjacent teeth with periapical infection [17]. Patients with RPI usually present with pain, tenderness, swelling, and fistula [20]. It is worth mentioning that RPI is different from peri-implantitis, where bone loss starts at the coronal part of the implant [11].

Previous studies addressed and reported the prevalence and possible causes of RPI. For example, Reiser and Nevins [21] reported the prevalence of RPI to be 0.26%, while Quirynen et al. [18] reported a 1.6% prevalence in the maxilla and 2.7% in the mandible. Although the prevalence of retrograde peri-implantitis is low, cases of RPI were said to be high when the implant sites were next to teeth with a previous history of root canal treatment or in patients with a previous periapical lesion (PAL) at the site of the implant or adjacent teeth [22,23,24].

Furthermore, RPI may be underreported as many clinicians are unaware of this type of lesion. Many authors suggested other risk factors for RPI, such as contamination of the implant surface, pre-existing bone disease, and periodontal diseases [25]. It is still unknown if these cases are strongly associated with the previous site of infected endodontically treated teeth or if this infection could also affect the adjacent teeth [26].

Therefore, more clinical studies are needed to investigate possible risk factors associated with RPI cases. This study aims to identify the incidence of RPI around implants placed at a single university dental hospital. The null hypothesis stated that endodontically treated teeth at the site or adjacent to dental implants have no effect on RPI incidence.

## 2. Materials and Methods

### 2.1. Experimental Design

This study was designed as a cross-sectional retrospective survey. Ethical approval was obtained from the College of Dentistry Research Center (CDRC) and the Institutional Review Board (IRB) at King Saud University, Riyadh, Saudi Arabia (Protocol # E-20-5187, approved on 16 September 2020) and was conducted in accordance with the Helsinki Declaration of 1975, as revised in 2013. Data were obtained by screening the dental electronic records of patients who received single-tooth implants placed at the university hospital between January 2016 and December 2020.

### 2.2. Inclusion and Exclusion Criteria

Inclusion criteria include (1) single implants with at least 3 months follow-up period after placement; (2) acceptable radiographs that show implants and adjacent teeth; (3) complete clinical and radiographic documentation.

Exclusion criteria include (1) implants placed elsewhere; (2) implants with no final restoration; (3) implants that do not have a radiograph of diagnostic quality; (4) implants with no complete data on the records.

### 2.3. Clinical Data Collection

For all implants included in the study, the following data was collected from patients’ electronic files:

1. Patients’ age and gender;
2. Self-reported medical history;
3. History of periodontal disease and treatment rendered. The history of periodontal disease was determined by checking the most recent periodontal chart, defining periodontal disease as the presence of at least 4 sites with clinical attachment loss (AL) ≥ 3 mm and a history of scaling and root planning [14]. The history of periodontal treatment was recorded;
4. Implant-related data, including date of implant placement, tooth number, tooth position (anterior vs. posterior), dental arch (maxillary vs. mandibular), the reason for extraction, the time elapsed before implant placement, type of implant placement (immediate, delayed), any other procedures that were done at the site (guided bone regeneration or socket preservation), implant type (straight, tapered), type of restoration (cement vs. screw-retained) and the number of units (single vs. multiple);
5. Endodontic status of the previous tooth at the implant site and the adjacent teeth.

### 2.4. Radiographic Evaluation

Periapical digital radiographs for implants and adjacent teeth were analyzed. Two board-certified endodontists evaluated the radiographs in a quiet room and dim lighting on a 15-inch computer screen using PPX (PowerPoint, Microsoft, Redmond, WA, USA) with the enhancement of resolution when needed. 

Teeth with endodontic treatment adjacent to implants were evaluated, and the following information was recorded:

- Periapical index score (PAI) [27], PAI scores were categorized as good (representing PAI scores of 1 and 2) and poor (representing PAI scores of 3, 4, and 5);
- Quality of endodontic treatment, described by adequate length 0–2 mm from the radiographic apex and adequate density with no evidence of missing canal. It was recorded as adequate or inadequate;
- Distance from the adjacent implant apex to the adjacent endodontically treated tooth was measured by drawing a perpendicular line from the tooth apex to the long implant axis. The distance was recorded and then categorized as equal/or less than 3 mm or more than 3 mm.

In addition, Implant sites were evaluated for the presence of radiolucency (RL) around the implant body and were scored as present or absent. Coronal bone loss characterized the cases of peri-implantitis that were not considered peri-implant RL.

If changes were noted around the implant, cases were placed for further evaluation as follows:

1. Review all radiographs for the implant site before, during, and after implant placement;
2. Evaluate clinical information related to implant and adjacent teeth treatment before, during, and after implant placement;
3. Any other treatment that was performed in the same area of interest

For establishing the diagnosis of RPI, the following criteria were used:

- Presence of periapical radiolucency around the implant that developed shortly after implant placement;
- The coronal portion of the implant achieves normal implant-to-bone contact;
- Patients may produce pain, tenderness, swelling, abscess, or suppuration based on electronic records.

### 2.5. Data Analysis

The data were transferred to a computer for analysis using Statistical Package for Social Sciences program for Windows (IBM SPSS Statistics Version 26, Chicago, IL, USA). Inter and intra-examiner reliability were calculated. Simple descriptive statistics such as frequency distributions and percentages were calculated for the study variables. Comparison between different variables was assessed using the chi-square test of independence or one-way ANOVA at 95% confidence (*p* ≤ 0.05).

## 3. Results

This study included 471 cases with 1036 implants, and 215 implants were included in the final analysis. All Implants sites were placed in one of the following groups:

Category A: implants placed next to endodontically treated teeth.

Category B: implant placed at the sites with previous endodontic involvement within 6 months of tooth extraction.

Category AB: implants that fulfill the criteria of groups A and B.

Category C: implants placed next to vital teeth and at a site with no previous endodontic treatment or a site that was allowed to heal for more than 6 months before implant placement if the extracted tooth was endodontically involved.

**Figure 1:**
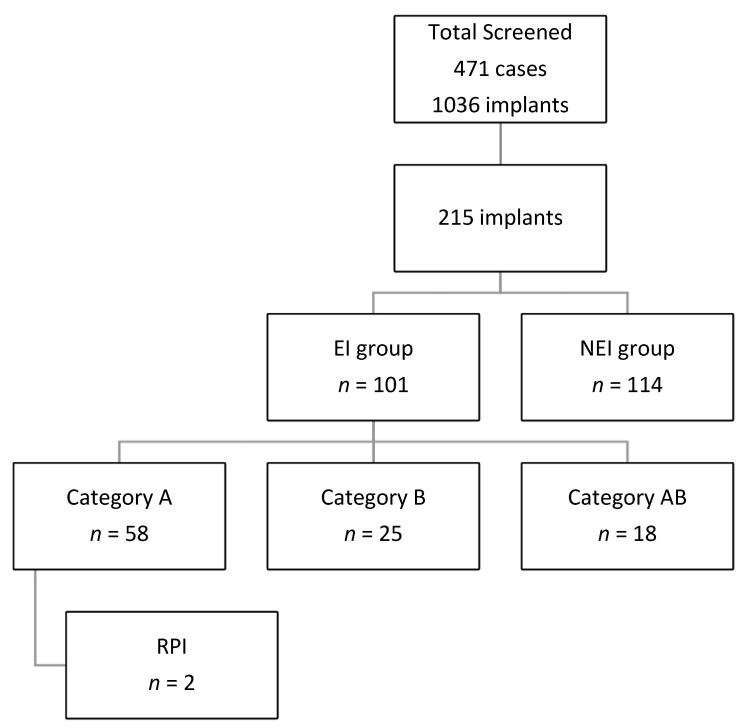
Methodology and case distribution. EI: endodontically involved, NEI: non-endodontically involved, RPI: retrograde peri-implantitis.

**Figure 2:**
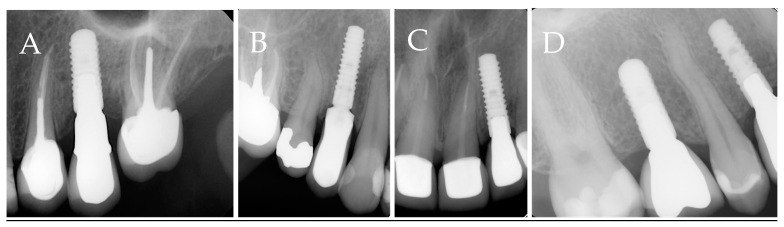
Radiographs representing different study groups. (**A**). Category A, implants placed next to endodontically involved teeth. (**B**). Category B, implants placed at a site with previous endodontic involvement within 6 months of extraction. (**C**). Category AB, implants that fulfill the criteria of groups A and B. (**D**). Category C, implants that are placed at a site with no previous endodontic involvement or a site with previous endodontic involvement that is allowed to heal for more than 6 months and next to vital teeth.

Categories A, B, and AB will be grouped into endodontic involvement (EI) groups. And category C will be considered the non-endodontic involvement (NEI) group. Data regarding the group distribution are shown in Figure 1. The radiographic representations of each study group are shown in Figure 2.

### 3.1. RPI Incidence and Its Relation to Demographic and Clinical Data

The demographics and clinical data for the study subjects are presented in Table 1. The mean age was 50.6 ± 13.4 years old, with equal proportions of males to females. The follow-up time after implant placement range from 13.3 to 216.9 weeks.

A total of 114 implants were in NEI (C) group. A total of 101 implants were grouped as EI, 58 in group A (27%%), 25 in group B (11.6%), and 18 (8.4%) in group AB. There was no statistically significant difference between the EI and NEI groups regarding factors studies, except gender and diabetic status (*p* = 0.001) Table 2. 

Reasons for teeth extraction were unrestorable (43.6%), endodontic-related complications (2%), and periodontal involvement (1%). Other teeth were extracted for unknown reasons (53.5%). Most implants were placed within 3–6 months after teeth extractions (45.5%). Immediate implant placement protocol was adopted in 15.8% of the cases. In comparison, the majority followed the delayed protocol (69.3%), and in 14.9% of the cases, the implant placement protocol was not stated clearly in the records. Bone augmentation procedures were used in 38.6% of the cases, and socket preservation was performed in 3%. Most implants were screw-retained (83.2%) and replaced single units (88.1%).

Details of the cases in groups A, B, and AB are presented in Table 3. There is a statistically significant difference between groups regarding gender (*p* = 0.009) and tooth position (*p* = 0.007).

### 3.2. The Proximity of Endodontic Teeth with the Periapical Lesions to Implants Increased the Risk of RPI

The weighted kappa value was calibrated before starting the study and calculated to be (k = 0.825) between the two endodontic examiners. A Kappa value was calculated after data collection, and the resultant was (k = 0.934).

Endodontically treated teeth next to implant sites in groups A and AB were evaluated by two endodontists for quality of endodontic treatment and PAI score. In addition, the distance between the endodontically treated teeth and the adjacent implant was measured. Most of the cases were scored as good with >3 mm distance between implants and adjacent teeth. There was no statistically significant difference between groups (*p* > 0.05) Table 4.

A total of 24 cases showed RL around the implant (11.2%), 22 among EI groups (17 in category A and five in category AB), and 2 among the NEI group. 

Only two sites from the whole sample (0.9%) were confirmed as RPI. Both are from the EI group and account for 2%. In addition, the two implants placed next to endodontically involved teeth (category A) represented 3.4% of that category. A total of 10 cases showed PAL around adjacent endodontically treated teeth, and two of them developed RPI, accounting for 20% incidence in cases where adjacent endodontically treated teeth have PAL. Both instances show proximity to the adjacent teeth (was ≤3 mm).

**Figure 3:**
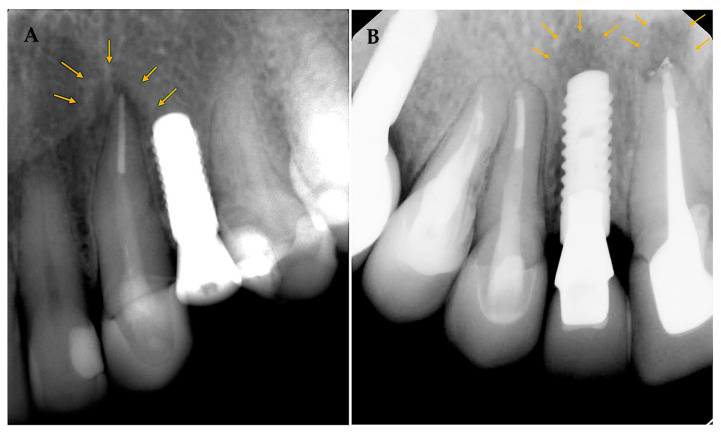
(**A**,**B**) show implant cases with RPI. Arrows to outline the periapical lesions in relation to teeth and adjacent implants.

Patients were brought for further clinical and radiographic evaluations for the cases diagnosed with RPI. Case 1 (Figure 3A) 56 years old female with a history of hypothyroidism (taking thyroxine). Tooth #24 was extracted, and the implant was placed more than 12 months after extraction (delayed implant placement protocol). Although the reason for extraction was not stated, RL developed around the implant body soon after implant placement and before loading. A diagnosis of asymptomatic reversible pulpitis was made for adjacent tooth #23, and endodontic treatment was initiated. No further treatment was performed, and the final restoration was placed after a few months. The implant was stable at the follow-up visit 4 years later. 

In case 2 (Figure 3B), 38 years old healthy female presented to replace an old fixed partial denture replacing teeth # 11–13 with 2 single crowns and an implant in the area of tooth #12. Both #11 and 13 had a previous root canal treatment. #11 had a PAL, and re-treatment was initiated before implant placement at the area of tooth #12. A few days later, the implant was placed at #12 (delayed implant placement protocol); soon after implant placement and before loading, RL developed around the implant body. Endodontic re-treatment was initiated on tooth #13. A few months later, the final implant restoration was placed. The implant was stable at the follow-up visit 4 years later. 

## 4. Discussion

The study aimed to evaluate the incidence of RPI around implants and possible risk factors. According to the current study, the results show no statistically significant difference between the groups in RPI incidence. Therefore, we accept the null hypothesis that endodontically treated teeth at the site or adjacent to the implant site do not affect the RPI incidence. However, the present study reported an increased incidence of RPI in implants next to endodontically involved teeth with PAL to 20% compared to other categories. This was statistically not significant and should be interpreted with caution.

Various factors can cause RPI, including endodontic treatment of the tooth at the site or adjacent to the implants, over-drilling or overheating during implant placement, bone perforations, or thinning at the apical part of the implant while preparing the implant site. In addition, several factors increase the incidence, such as endodontic infections, an implant placed close to the infection site and the presence of PAL on adjacent teeth [14,19].

The overall Incidence of RPI in the present study is 0.9%, with 3.4% occurring when adjacent teeth are endodontically involved. The reported Incidence of RPI in literature is based on retrospective analysis, case series, and case reports with significant variations. In recent retrospective studies, an incidence of 0.34% was reported at sites with previous infections due to endodontic or periodontal diseases. The authors declare that previous infection and residual microorganisms at the implant site could be reactivated during the implant site preparation, causing RPI [8,28]. Lefever et al. [23] showed a much higher incidence, with around 14% of the cases adjacent to teeth with PAL showing RPI. Another study showed that sites with previous endodontic failure and apical surgery show RPI in 20% of the cases [29]. In the present study, the sites with previous endodontic involvement did not show an increased risk of RPI. The presence or absence of periapical changes around the extracted tooth could explain these results. Quirynen et al. [18] and Di Murro et al. [8] reported that RPI is correlated with endodontic failure or pathology at the extraction site. However, no analysis of the teeth at the implant sites was performed, as no cases show RPI in that category. In addition, only 2% of the teeth were extracted due to endodontic reasons among cases.

Similar to the current study findings, studies reported an association between the status of the adjacent teeth and RPI. For example, Zhou et al. [22] showed a 7.8% incidence of implants placed next to teeth with recent endodontic treatment. Therefore, the authors suggested allowing enough healing time for adjacent teeth after endodontic treatment and increasing the distance between the implant apex and adjacent teeth. On the other hand, the presence of PAL at the adjacent teeth has been associated with a 3.7% [30] to 25% incidence [23].

Both cases with RPI in this study presented implants adjacent to endodontically treated teeth. In addition, these teeth had a poor PAI score (4) and showed proximity to the site on the implant (<3 mm). PAL and the tooth to implant distance have been reported as risk factors in previous studies [22,23,30]. On the other hand, about 20% of the cases analyzed presented poor PAI scores, and 15% showed high proximity to adjacent teeth without any changes around the implants. Therefore, assessing all the findings at the future implant site is essential, as there could be a combination of factors that increase the risk of RPI. 

During radiographic analysis, 24 cases presented with possible RPI. Careful cases assessment excluded most of them (22 cases), including cases where PAL/scar was shown before implant placement and remained unchanged afterward or PAL was detected immediately after implant placement which could suggest the drilling of perforation. This could help identify RPI cases due to endodontic involvement and contribute to the low incidence reported in the present study.

It is important to note that there were more diabetic patients among the EI group compared to the NEI group. The effect of diabetes mellites on healing is well documented due to the glycated hemoglobin by-products’ effect on different body organs. This effect is more pronounced in non-controlled cases. A recent systematic review [31] reported a strong correlation between the presence of PAL on endodontically treated teeth in diabetic patients compared to control. However, in the present study, most endodontically treated teeth presented good PAI among people with diabetes. Furthermore, the cases with RPI in the current research were non-diabetics.

Sarmast et al. presented a decision tree to help diagnose and manage RPI cases. It was suggested that treatment rendered should be based on patient symptoms and the presence of endodontic involvement. It can be as simple as nonsurgical endodontic treatment or implant removal in advanced cases [24]. Early diagnosis and management of the case prevent further infection and bone loss around the implant. Both cases with RPI in the present study were diagnosed in the first few weeks after implant placement and before prosthetic leading. The cases presented were managed successfully by initiating endodontic treatment or re-treatment. The follow-up visit almost 4 years post-implant placement shows successful implants on clinical and radiographic examination. This agrees with a previous case series that showed RPI could be successfully managed by nonsurgical endodontic treatment in cases of endodontic infection on adjacent teeth [12].

## 5. Limitations

There is some limitation to this study, including its retrospective nature, small sample size, and a small number of cases identified with RPI, that preclude further analysis. The effect of patients’ medical status on the Incidence of RPI could be assessed as the number of cases was low.

However, the rigorous selection criteria of the included cases and the exclusion of the cases with missing or no precise data add strength to this study. In addition, the distribution of the cases to different groups according to case history and inclusion of the NEI group allow valid comparisons. Last, clinical and radiographic follow-up for the cases diagnosed with RPI proves that implant treatment can be successful with nonsurgical endodontic treatment.

## 6. Conclusions

Despite the present study’s low Incidence of RPI, careful site assessment before implant placement can reduce the risk of implant complications. Endodontically treated teeth with PAL close to an implant site could contribute to RPI. Therefore, clinicians are strongly encouraged to evaluate adjacent teeth and allow enough healing time in cases of infection before implant placement. Endodontic treatment was sufficient in these cases to stabilize the implant at the follow-up visit.

## Figures and Tables

**Table 1:** Demographics and clinical data.

	Mean ± SD
Age	50.6 ± 13.4 years old
Implant follow-up time	66.7 ± 41.6 weeks
	***n* (%)**
Gender	109 (50.7%) M, 106 (49.3%) F
Diabetes Mellitus	20 (9.3%)
Smoking	3 (1.4%)
History of Periodontitis	6 (2.8%)
Tooth position	184 (85.5%) Post, 31 (14.4%) Ant
Dental arch	127 (59.1%) Max, 88 (40.9%) Mand

M: males, F: females.

**Table 2:** Comparison of demographic and clinical data between the two groups.

	EI Group *n* = 101	NEI Groups *n* = 114	*p*-Value
Mean ± SD			
Age	51.1 ± 12.4 years old	50.1 ± 14.3 years old	>0.05
Implant follow-up time	62.9 ± 38.1 weeks	70.1 ± 44.2 weeks	>0.05
***n*** **(%)**			
Gender	39 (38.6%) M, 62 (61.4%) F	70 (61.4%) M, 44 (38.6%) F	=0.001 *
Diabetes Mellitus	17 (16.8%)	3 (2.6%)	=0.001 *
Smoking	2 (2%)	1 (0.9%)	>0.05
Tooth position	86 (85.1%) Post 15 (14.9%) Ant	98 (86%) Post 16 (14%) Ant	>0.05
Dental Arch	59 (58.4%) Max 42 (41.6%) Mand	68 (59.6%) Max 46 (40.4%) Mand	>0.05
RPI	2 (2%)	0	>0.05

* statistically significant difference was found between the 2 groups in gender and diabetic status. EI: endodontically involved, NEI: non-endodontically involved, RPI: retrograde peri-implantitis.

**Table 3:** Comparison of demographics and clinical data between categories A, B and AB.

	A = 58	B = 25	AB = 18	*p*-Value
	Mean ± SD			
Age	50.1 ± 11.5 years old	52.6 ± 15 years old	51.9 ± 11.5 years old	>0.05
Implant follow-up time	63.7 ± 40.2 weeks	58.9 ± 32.9 weeks	65.8 ± 39.9 weeks	>0.05
	***n* (%)**			
Gender	16 (27.6%) M, 42 (72.4%) F	16 (64%) M, 9 (36%) F	7 (38.9%) M, 11 (61.1%) F	=0.009 *
Diabetes Mellitus	11 (19%)	1(4%)	5 (27%)	>0.05
Smoking	1( 1.7%)	0	1 (5.6%)	>0.05
Tooth position	53 (91.4%) Post 5 (8.6%) Ant	21 (84%) Post 4 (16%) Ant	12 (66.7%) Post 6(33.3%) Ant	=0.007 *
Dental Arch	32 (55.2%) Max 26 (44.8%) Mand	17 (68%) Max 8 (32%) Mand	10 (55.6%) Max 8 (44.4%) Mand	>0.05
RPI	2 (2%)	0	0	>0.05

* statistically significant difference was found between the three categories in regard to gender and tooth position. SD: standard deviation, RPI: retrograde peri-implantitis.

**Table 4:** Endodontic evaluation of teeth adjacent to implants in categories A and AB.

		Category A *n* (%)	Category AB *n* (%)	*p*-Value
PAI	Good	48 (82.8%)	12 (66.7%)	>0.05
	Poor	10 (17.2%)	6 (33.3%)	
Distance between Implant and EI tooth	≤3 mm	6 (10.3%)	6 (33.3%)	>0.05
	>3 mm	52 (89.7%)	12 (66.7%)	

No statistically significant difference between the two groups in endodontic evaluation. PAI: periapical index score, EI: endodontically involved.

## Data Availability

The data presented in this study are available on request from the corresponding author.

## Abbreviations

RPI	Retrograde peri-implantitis
PAL	periapical lesion
PAI	Periapical index
RL	radiolucency
EI	endodontic involvement
NEI	non-endodontic involvement
